# Combining Microbial Culturing With Mathematical Modeling in an Introductory Course-Based Undergraduate Research Experience

**DOI:** 10.3389/fmicb.2020.581903

**Published:** 2020-11-06

**Authors:** Robert E. Furrow, Hyunsoo G. Kim, Samah M. R. Abdelrazek, Katherine Dahlhausen, Andrew I. Yao, Jonathan A. Eisen, Mark S. Goldman, John G. Albeck, Marc T. Facciotti

**Affiliations:** ^1^Department of Neurobiology, Physiology, and Behavior, Center for Neuroscience, University of California, Davis, Davis, CA, United States; ^2^Graduate Group in Microbiology, University of California, Davis, Davis, CA, United States; ^3^Genome Center, University of California, Davis, Davis, CA, United States; ^4^Department of Anatomy, Physiology, and Cell Biology, School of Veterinary Medicine, University of California, Davis, Davis, CA, United States; ^5^Molecular Prototyping and BioInnovation Lab, Department of Biomedical Engineering, University of California, Davis, Davis, CA, United States; ^6^Department of Evolution and Ecology, University of California, Davis, Davis, CA, United States; ^7^Department of Medical Microbiology & Immunology, University of California, Davis, Davis, CA, United States; ^8^Department of Ophthalmology and Vision Science, University of California, Davis, Davis, CA, United States; ^9^Department of Molecular and Cellular Biology, University of California, Davis, Davis, CA, United States

**Keywords:** CURE, microbial diversity, microbiome, microbial culturing, mathematical modeling, education, logistic growth curve analysis

## Abstract

Quantitative techniques are a critical part of contemporary biology research, but students interested in biology enter college with widely varying quantitative skills and attitudes toward mathematics. Course-based undergraduate research experiences (CUREs) may be an early way to build student competency and positive attitudes. Here we describe the design, implementation, and assessment of an introductory quantitative CURE focused on halophilic microbes. In this CURE, students culture and isolate halophilic microbes from environmental and food samples, perform growth assays, then use mathematical modeling to quantify the growth rate of strains in different salinities. To assess how the course may impact students’ future academic plans and attitudes toward the use of math in biology, we used pre- and post-quarter surveys. Students who completed the course showed more positive attitudes toward science learning and an increased interest in pursuing additional quantitative biology experiences. We argue that the classroom application of microbiology methods, combined with mathematical modeling using student-generated data, provides a degree of student ownership, collaboration, iteration, and discovery that makes quantitative learning both relevant and exciting to students.

## Introduction

The American Association for the Advancement of Science and the National Research Council have each called for renewed undergraduate education efforts to build broadly applicable biology research skills (National Research Council, 2003, 2009; American Association for the Advancement of Science, 2011). One report, *Vision and Change* (American Association for the Advancement of Science, 2011), laid out influential and ambitious goals for reforming undergraduate biology education, encouraging the integration of core concepts and competencies throughout the curriculum. Several of these competencies are quantitative, including the ability to use quantitative reasoning and the ability to apply modeling and simulation.

However, quantitative material can be challenging to introduce to biology-interested students early in their undergraduate career. Students enter college with a wide range of mathematics skills (Treisman, 1992; Agustin and Agustin, 2009; Sonnert and Sadler, 2014), and experiences in traditional introductory courses like calculus might lead some students to leave STEM (Ellis et al., 2016). In addition, many undergraduate biology students may also have unfavorable emotions about math (Wachsmuth et al., 2017). These emotions can translate to poor performance in math-related coursework (Ma and Kishor, 1997).

One way to address these challenges is by integrating math and biology coursework at multiple points along an undergraduate curriculum (Bialek and Botstein, 2004; Chiel et al., 2010; Depelteau et al., 2010; Duffus and Olifer, 2010; Miller and Walston, 2010; Aikens and Dolan, 2014; Eaton and Highlander, 2017). In our experience, however, few introductory biology lab courses emphasize the breadth of quantitative skills commonly used in biology research. We propose that introductory course-based undergraduate research experiences (CUREs) may be a valuable early part of this type of integrated curriculum, given their potential positive effects on student learning and attitudes.

CUREs are natural candidates to promote quantitative learning and build positive attitudes toward math among biology-interested students. These courses engage students in the practice of research from within the classroom, emphasizing peer collaboration and iterative approaches to the research process while students use modern scientific practices to address novel, broadly relevant research questions (Auchincloss et al., 2014). Student participation in CUREs can benefit student learning as well as persistence in STEM and attitudes toward science (Brownell et al., 2012; Jordan et al., 2014; Olimpo et al., 2016; Rodenbusch et al., 2016; reviewed by Dolan, 2016), and these courses may provide an avenue toward creating a more inclusive academic environment (Bangera and Brownell, 2014). Several recent CUREs have included quantitative learning outcomes and found student benefits (Brownell et al., 2015; Kirkpatrick et al., 2019; Murren et al., 2019), although these courses typically focus more on data and figure interpretation than on mathematical modeling.

In this study we outline an introductory quantitative biology CURE that combines microbial culturing and genomic DNA isolation with modeling and quantitative characterizations of growth rate. We assessed student attitudinal gains using several published instruments (Andrews et al., 2017; Lopatto et al., 2008; Shaffer et al., 2010), as well as short-answer questions related to students’ future course and career plans. We sought to answer three questions.

1. Would this quantitative biology CURE increase students’ interest in and perceived utility of using math in biology?
2. Would this course help students develop more positive attitudes toward science learning?
3. Does this course influence student plans for future quantitative courses or careers?

We hypothesized that this course might increase students’ desire to pursue future quantitative biology experiences by building more positive attitudes toward science learning and toward using math in biology. Assessing changes in student attitudes toward math in biology proved difficult due to strongly positive initial attitudes in this self-selected population. However, we find some evidence of positive changes in student attitudes toward learning science, as well as increased student interest in pursuing future quantitative experiences.

## Materials and Methods

### Developing the Course Structure and Subject

Here we outline the process we followed in creating a quantitative biology CURE. We developed the course to help students build quantitative skills that are commonly used in biology research. To that end, we informally surveyed the laboratory and quantitative skills used in local microbiology research labs. We identified commonly used lab skills including microbial culturing, microscopy, and spectrophotometry, which integrated with quantitative skills like calculations of concentration and dilution factors as well as mathematical modeling of growth curves. We chose to modify an existing workflow that is commonly used in undergraduate research on the microbiome (Dunitz et al., 2015). In this workflow, students culture microbes from almost any environmental sample, generate isolates from the sample(s), and use growth curves of the isolates to quantify aspects of the organism’s biology. The workflow was initially piloted as a less quantitatively oriented seminar course, using environmental samples ranging from nectar (Dahlhausen, 2018) to abalone (Vater et al., 2016) to koala feces (Coil, 2017). These courses and other CUREs have been discussed by Vater et al. (2019). In our more quantitatively focused version of this microbial isolation approach, the specific taxa play a minimal role in shaping the course learning goals, teaching methods, and assessments.

The current iteration of this CURE focuses on culturing, isolating, and quantitatively characterizing the features of halophiles (Rodriguez-Medina et al., 2020). Halophiles are a category of microorganisms that thrive in hypersaline conditions from sea salinity to saturation. These organisms span all three domains of life and can be found in diverse global environments including hypersaline soils, lakes, solar salterns, deep salt mines, and natural brines in coastal and submarine pools (DasSarma and DasSarma, 2015; Torregrosa-Crespo et al., 2017). Some halophiles are known to be polyextremophiles that are capable of tolerating and thriving not only in hypersaline environments, but also in settings with high pH, large amounts of sun radiation, and/or low water or nutrient availability. These harsh conditions have allowed halophiles to adapt unique biochemical pathways of interest in both basic and applied research realms (Becker et al., 2014; Torregrosa-Crespo et al., 2017). Several products derived from these pathways are of particular commercial interest, including but not limited to: polyhydroxyalkanoates (plastics industry), amylases (biofuel industry), proteases (laundry detergent), beta-carotene (food additive), and glycerol (cosmetic industry) (Yin et al., 2015; Amoozegar et al., 2017).

The ability of halophiles to thrive in harsh conditions also makes them practical to use in the classroom. Because hypersaline growth conditions are inherently inhibitory to non-halophiles, halophilic culturing is more forgiving to small lapses in sterile technique, allowing students to successfully isolate pure cultures even if they are inexperienced in the laboratory. This is well-suited for introducing lab work and microbial culturing to first-year students. Although halophiles have a relatively long doubling time, a single weekly laboratory allows enough time for culture growth between each session.

### Learning Outcomes and Course Overview

There are three main learning outcomes for the course: (1) students will be able to plan and perform the process of microbial culturing and genomic isolation, (2) students will be able to fit population growth models to microbial growth curve data, plot results, and compare the quality of fit among competing models, and (3) students will build interest and confidence in using quantitative skills in biology.

The structure of our 10-week quantitative biology CURE includes 1 weekly 3-h wet lab session, currently offered in the Molecular Prototyping and BioInnovation Lab at UC Davis, as well as a 1-h weekly lecture held in a traditional classroom. The lectures cover the quantitative theory associated with the hands-on research experience and connect this theory to the lab applications (Supplementary File S2). Students also complete a weekly homework that combines a lab write-up with quantitative problem-solving. As the course transitions into student projects, student learning is assessed with an initial project proposal, a final written report, and an oral group presentation.

The lab starts with an introduction to formal campus and site-specific lab safety training. We require all students to successfully complete the University of California “Fundamentals of Laboratory Safety Training,” an online course required for everyone who works in labs on campus. We explain to the students that this training certifies them to work in faculty research labs on campus. The site-specific training highlights that the workspace is used outside of class hours to host active student research projects (i.e., they are working in a “real” research space and not just a classroom and thus that we expect them to be aware of those activities and associated hazards as they work). We emphasize this training to provide a solid foundation in safety, but also to establish a classroom environment in which students feel like they are doing authentic research.

Following the safety training, an introductory activity on pipetting, mixing, and measurement teaches techniques and orients students to the various instruments and supplies in the lab required for the course. These hands-on exercises are complemented with lecture and homework in which students develop their understanding of measurement and sources of error. For example, in one activity students repeatedly pipette and weigh an identical volume of solution. During lecture students had previously learned about accuracy and precision and how these can be quantified, and on the homework students use the programming language R (R Core Team, 2019) to calculate these quantities for their self-collected measurements. This pattern of linked lecture, lab, and homework continues throughout the quarter as each student collects (or selects previously collected) environmental samples, progresses through microbial isolation, and phenotypically characterizes the isolates.

In 2018, students worked with different table salts available from local supermarkets, as well as from environmental samples collected at the salt flats of Cabo Rojo, Puerto Rico. In 2019, students self-collected local samples from soil, water, or salty foods. Students then practice microbial culturing and isolation, using their samples to start cultures. For this course, we use Halobacterium medium 372 (DSMZ, 2007) as the base medium and vary the concentrations of NaCl. By using media with multiple salinities, we can potentially culture a broader diversity of microbes from the samples. All cultures are grown in either a shaking (liquid culture) or static (agar plate) incubator at 37°C. Plates that grow colonies within 7 days are transferred to a 4°C refrigerator to pause growth. To prepare pure cultures, students make phenotypic observations of a single colony of interest, select cells from the colony to inoculate a new liquid culture, and prepare microscope slides to make observations on cell size and shape using a standard compound microscope equipped with phase contrast optics.

**FIGURE 1:**
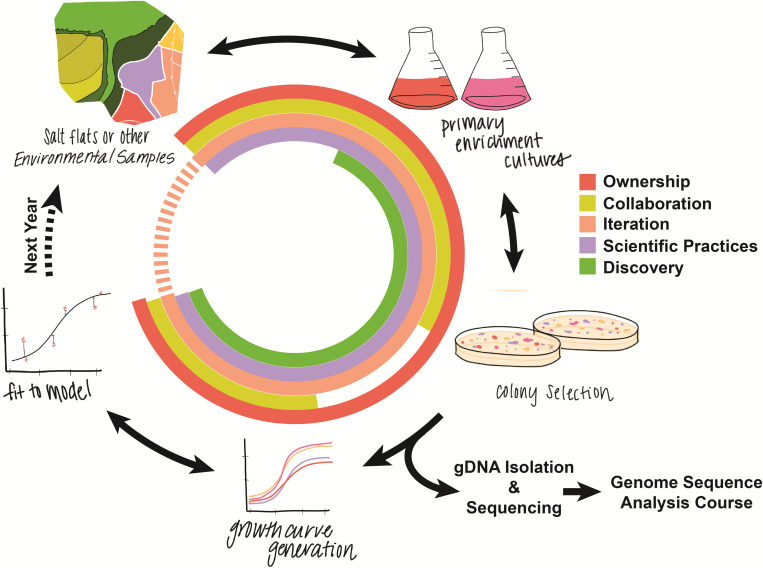
Graphical overview of the course structure. Colors in the circle correspond to different core elements of course-based undergraduate research experiences. Arrows in both directions represent places where students can iteratively repeat and revise, for example re-plating primary enrichment cultures to look for novel colonies or fitting additional models to growth curves.

With pure liquid cultures in hand, students focus on measuring and modeling microbial growth. Using a spectrophotometer, students measure the growth of each isolate at multiple salinities to quantitatively characterize salt tolerance. Students then use the programming language R (R Core Team, 2019) to fit logistic growth models to their data, explore variants of the models, and decide on the model that best explains the patterns they observe. The course culminates with students performing genomic DNA isolation and shipping off the gDNA for whole genome sequencing. These novel data are then used in a spring quarter follow-up CURE in which students continue to build quantitative skills as they apply bioinformatic and statistical techniques for comparative genomic analyses (Figure 1). Supplementary Files S2–S7 present examples of additional curricular materials: a weekly course schedule, an example lab protocol, an R notebook for a computational lab, the associated RData file for the computational lab, the prompt outlining the students’ final project, and an assignment that provides students with practice in writing up their final project report.

Iteration is considered a fundamental feature of CUREs. In this course, the steps described above may be iterated in practice by allowing students to revise and redo experimental or analytical steps. For instance, students have repeated opportunities to perform new isolations, select a new sample, redo growth measurements, and revise their quantitative growth models. Figure 1 outlines the steps in the research process for this course, highlighting how the activities relate to iteration and other key features of CUREs, including peer collaboration, discovery, and scientific practices (Auchincloss et al., 2014).

We emphasize that students own every step of this project. They select their samples and media, maintain their cultures, and make their own decisions about how to revise their models. Because project ownership may be a critical mediator of students’ overall benefits in CUREs (Corwin et al., 2018), the course aims to develop students into independent lab practitioners who are progressing their own projects.

### Assessment of Student Attitudinal Gains

This work was approved by the University of California, Davis Institutional Review Board (protocol #1314250). We surveyed students who enrolled in the halophile CURE in the fall quarters of 2018 and 2019. Because this was an elective course that did not satisfy any major-specific requirements, this sample is likely biased toward students who are motivated to pursue laboratory and quantitative experience. As a basis for comparison, we recruited a non-overlapping group of survey respondents in the University Honors Program who had a biology-related major, both because many of our students were in the University Honors Program and because we hypothesized that other University Honors students may also be motivated to pursue research-oriented laboratory and quantitative experience. Some students who enrolled in the CURE were also in the University Honors Program (12 out of 16 students in 2018 and 5 out of 17 students in 2019). This comparison group was surveyed only in 2018, as the set of available comparison students in 2019 mostly overlapped with those from 2018.

Students completed an initial (pre) survey online during the first week of the quarter and completed an end-of-quarter (post) survey during the final weeks of the quarter. Students in the CURE completed the surveys during class time, while the comparison group completed their surveys at their own pace, outside of class. Authors JGA and MTF were co-instructors for the CURE in 2018, and authors JGA and REF were co-instructors in 2019. REF performed all data analysis on the anonymized student responses. The surveys contained multiple choice questions used in both years, and short response questions that were added in 2019.

Survey data can be contaminated by participants who provide inaccurate responses to questions. We ensured that students had read the questions by including one 5-point Likert-scale question that stated “We use this question to discard from the survey people who are not reading the questions. Please select Agree (not Strongly agree) for this question to preserve your answers.” We excluded from our analysis any survey responses with a choice other than “Agree” for this question for both the initial and end-quarter surveys. This excluded two students (out of 52 respondents). One additional student was excluded due to an incomplete survey.

#### Final Student Data

In total, we analyzed consenting, quality-controlled, paired pre and post responses from 16 CURE students in 2018 (94% of enrolled students), 17 CURE students in 2019 (77% of enrolled students), and 16 students in the comparison group (11% of the students initially emailed at the start of the quarter). Due to the small sample size of this study, we do not report specific demographic information or attempt to analyze the effect of demographics on student outcomes. Approximately 60% of students in both the CURE and comparison groups were female. 82% of students in the CURE were in their first or second year, as were all students in the comparison group. Student majors varied widely in both groups. Due to the range of years and majors, it is unlikely that a substantial proportion of either the CURE or comparison group shared any particular other courses. 17 of the 33 CURE students were in the University Honors Program, as were all 16 students in the comparison group. To have a larger sample size, data from both years of the CURE were merged and analyzed together. We note that patterns of student responses in the CURE group were similar in both years (Supplementary Figure S1). Our analysis focused on comparing survey responses on the end-of-quarter (post) survey to the responses on the initial (pre) survey, and contrasting these patterns between the CURE and comparison groups.

#### Student Attitudinal Changes

To assess changes in student attitudes, we used two previously created assessment instruments. The Math-Biology Values Instrument (Table 1; Andrews et al., 2017) assesses student attitudes toward using mathematics in biology, and is grounded in expectancy-value theory of achievement motivation and performance (Eccles et al., 1983; Wigfield and Eccles, 2000; Eccles and Wigfield, 2002). This theory posits that student achievement depends both on a student’s confidence of success and the value they see in completing a task (Wigfield and Cambria, 2010; Corwin et al., 2018). This instrument consists of 11 questions that assess three underlying constructs related to student perceptions of using math in biology: ***interest*** in, ***utility*** of, and ***cost*** of taking biology courses that incorporate math (Table 1). To analyze student changes in their math-biology values, we created a subscore for each construct, averaging across all relevant questions. Finally, we used a Mann-Whitney *U*-test to compare the mean change in each subscore between the comparison group and the CURE students.

**TABLE 1:** The Math-Biology Values Instrument questions.

**Item text**	**Construct**
Using math to understand biology intrigues/would intrigue me.	Interest
It is/would be fun to use math to understand biology.	
Using math to understand biology appeals/would appeal to me.	
Using math to understand biology is/would be interesting to me.	
Math is valuable for me for my life science career.	Utility
It is important for me to be able to do math for my career in the life sciences.	
An understanding of math is essential for me for my life science career.	
Math will be useful to me in my life science career.	
I have/would have to work harder for a biology course that	Cost
incorporates math than for one that does not.	
I worry/would worry about getting worse grades in a biology	
course that incorporates math than one that does not.	
Taking a biology course that incorporates math	
intimidates/would intimidate me.	

**All questions were framed with a 7-point Likert scale, asking “For each of the statements in this set please rate your agreement with the item in question.” Respondents could answer “Strongly disagree,” “Disagree,” “Somewhat disagree,” “Neither agree nor disagree,” “Somewhat agree,” “Agree,” or “Strongly agree,” which we translated to a 1 through 7 scale. These questions assess three underlying constructs, labeled Interest, Utility, and Cost.**

To evaluate student attitudes toward science learning and the scientific process, we used a subset of questions from the “Your opinions about yourself and about science” section from the CURE survey (Lopatto et al., 2008; Shaffer et al., 2010). The questions are summarized in Table 2. To avoid the problem of multiple comparisons that arise when testing the statistical significance of many individual questions, we relied on previous efforts that have assessed underlying relationships between questions. Prior analysis of this survey used factor analysis to identify the internal structure of these questions, finding two distinct constructs that are each assessed by multiple questions (Perera et al., 2017), and authors REF and MSG have noted similar correlations in student responses for these questions on a different study of 1,800 student responses (Furrow, Caporale, and Goldman, unpublished). We created two subscores using the relevant questions from our survey and used Mann-Whitney *U*-tests to assess the statistical significance of differences among groups for each subscore. We label one construct Personal Value, following the nomenclature of Perera et al. (2017). The other construct is based on a smaller subset of the questions found to be correlated in prior work; because the questions all focus on science learning, we label the construct Science Learning. These questions are negatively framed, with greater disagreement expressing more positive attitudes toward science learning. Any quantitative analyses of student changes for this subscore are reverse-coded to assign higher values to more positive attitudes.

**TABLE 2:** CURE Science Attitudes questions and their alignments with previously established constructs.

**Item text**	**Construct**
Even if I forget the facts, I’ll still be able to use the	Personal value
thinking skills I learn in science.	
I get personal satisfaction when I solve a scientific	
problem by figuring it out myself.	
I can do well in science courses.	
Explaining science ideas to others has helped me	
understand the ideas better.	
There is too much emphasis in science classes on figuring things out for yourself.	Science Learning
I wish science instructors would just tell us what we need to know so we can learn it.	
Science is essentially an accumulation of facts, rules, and formulas.	
*To be successful in biology, I need to be able to perform*	**Calculations**
quantitative calculations.	
*Mathematical models are useful for biology research.*	**Models**

**All questions were framed with a 5-point Likert scale, asking “For each of the statements in this set please rate your agreement with the item in question.” Respondents could answer “Strongly disagree,” “Disagree,” “Neutral,” “Agree,” or “Strongly agree,” which we translated to a 1 through 5 scale. Italicized items were newly created for this survey. Items in the Science Learning construct were reverse-scored for quantitative analysis, as they are negatively framed.**

This section of the survey also included two additional statements posed in the same format (Table 2): “*Mathematical models are useful for biology research*” and “*To be successful in biology, I need to be able to perform quantitative calculations*,” hereafter labeled as the Models and Calculations questions, respectively. These questions measure student perceptions of the utility of specific mathematical approaches in biology.

#### Changes in Students’ Future Course and Career Plans

To assess students’ future course and career plans related to quantitative biology, we asked additional qualitative questions in the survey for the 2019 iteration of the CURE. In the initial survey we asked: “*Do you have any plans to pursue future courses and/or a career in quantitative biology? Please briefly explain why or why not.*” In the end-of-quarter survey, we asked a matched short-answer question: “*Has this course changed your plans for pursuing future courses and/or a career in quantitative biology? If so, how?*” These end-of-quarter responses were then organized by theme (Table 3).

**TABLE 3:** Summary of student responses to the short-answer survey questions about future academic plans related to quantitative biology.

**Theme (# students)**	***[Pre] Do you have any plans to pursue future courses and/or a career in quantitative biology? Please briefly explain why or why not.***	***[Post] Has this course changed your plans for pursuing future courses and/or a career in quantitative biology? If so, how?***
Increased interest (6)	“As a biomedical engineer, I plan to focus on bioinformatics/bioimaging during my college career. Utilizing programming and mathematical modeling, I hope to gain more insight to human physiological processes while learning biology.”	“I initially planned on pursuing just a major in Biomedical Engineering, but the content in this course has encouraged me to pursue a minor in Computational Biology. I sincerely enjoyed the mathematical applications related to biology and wish to pursue further research into mathematics in life sciences overall.”
	“Yes! I am planning on pursuing a federal position as a government scientist in general scientific concerns, possibly, and most likely involving quantitative biology.”	“This course has changed my plans career-wise in that I am much more comfortable considering a pursuit in quantitative biology.”
	“Yes, quantitative biology is something that I have only recently learned more about and would definitely like to take more coursework if possible.”	“I am definitely more inclined to investigating the possibilities that are available in quantitative biology. I was hoping to explore this interest further as I enrolled in this class and am happy to say that I definitely found the passion that I was expecting.”
	“I’m not sure yet, but I wish to be a researcher in the future because it is interesting.”	“Yes I might take more bio modeling courses”
	“I have no concrete plans, but I am taking this class to see whether such a career would interest me.”	“I’m still undeclared and don’t yet have any idea what my career will be. The course has definitely made me more interested in quantitative biology though. I will probably take BIS23B, and would consider taking more quantitative biology courses in the future.”
	“Yes, because maths is making stuff more interesting and as a comp sci major i like maths	“it convinced me that quantitative biology is interesting”
Career clarification (2)	“I am unsure at the moment as I have never had to previously incorporate much mathematics in my biology courses before. I really enjoy learning and trying to understand the difficult concept in biology, but do find math quite intimidating. Though I know that math is fundamental for all science-related fields, I do not think I can see myself pursuing any careers in quantitative biology in the future.”	“Overall, this course has helped me gain a better understanding as to how research and biology in certain experiments are best explained through the use of mathematical models. Though math is a challenging and often daunting subject for me, I do believe that it is essential to understanding and applying a bit of it into the science world. I [am] not sure if I can succeed in a career centered around quantitative biology.”
	“While I’m not completely sure whether or not I will pursue a career in quantitative biology, I know for sure that I will continue taking quantitatively based biology classes and therefore I am pretty certain that I will most likely end up with a career in quantitative biology.”	“It has definitely made me reconsider what exactly I want to do. I am still not sure about what I want to do or what to pursue in the future but this class has given me valuable insight.”
Sustained interest (5)	“Yes as I am a genetics major and am wildly interested in going into research during my university career and beyond”	“I’m still interested in pursuing a career in genetics and genomics, this course has helped me basically see and understand the power of quantitative analysis in biology more so than I did before without experience”
	“I plan to pursue future courses in quantitative biology because biology is starting to become a data-driven science and it is important that undergraduates like myself are able to deal with this trend.”	“I plan on taking BIS 23B and extra math courses to help understand other biological phenomena.”
	“I am applying for graduate schools in the field of biomedical informatics and computational biology.”	“No, I already planned to pursue a career in q bio”
	“Yes, it gives me a chance to apply what I know rather than soaking up information and not being able to do anything with it.”	“No”
	“I would like to do the quantitative biology major because I am interested in both cs and biology”	“After taking this course, I would like to take BIS 23B in the spring (and perhaps BIS 20Q in the winter).”
Unclear (4)	“Maybe. I’m thinking about researching epigenetics for medical applications. I know that bioinformatics is important and that big data is becoming more prevalent in genetics. I would say quantitative biology isn’t my goal, but may be where I end up.”	“Honestly, the coding component was kind of a shock. It’s tough at first but rewarding once you finally get it.”
	“Yes, I plan to work in a research field in Genetics.”	“I really wanted to take a class that gave me an experience of what a lab actually is like, this class did that and was really enjoyable.”
	“I am unsure of whether I want to pursue future courses and/or a career in quantitative biology because I am still unsure of what such courses/careers would entail.”	“It hasn’t really changed much of my plans.”
	“At the moment, no. I took this course to gain more lab experience.”	“Plan on doing research”

**Student pre and post responses are aligned by row. The initial (pre) survey asked students about future plans, and the end-of-quarter (post) survey asked if their plans had changed. These questions were asked only to the CURE students enrolled in 2019 (17 respondents in total). We categorized the paired pre and post responses into four codes: “Increased interest,” “Career clarification,” “Sustained interest,” and “Unclear.” Responses that directly mentioned an increase in confidence or interest in future courses or careers in quantitative biology were coded “Increased interest.” Responses that discussed insight or understanding about what this work looks like were coded “Career clarification.” The “Sustained interest” code was used for responses that didn’t explicitly mention any gain in interest, but expressed similarly positive plans in both pre and post. Responses that did not clearly address the survey questions were coded as “Unclear.”**

## Results

### Math-Biology Values Had Minimal Change

**FIGURE 2:**
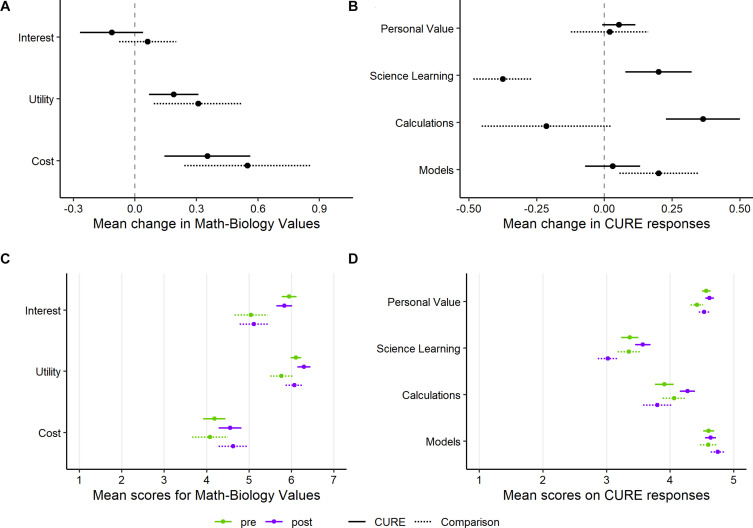
Mean change (post score minus pre score) in student response between initial (pre) and end-of-quarter (post) survey, as well as mean pre and post scores, for several assessment instruments. **(A)** Change for each of the three constructs assessed by the Math-Biology Values Instrument, averaged over the questions for each construct. **(B)** Change for two constructs assessed by questions on the CURE survey, as well as two additional questions specific to quantitative thinking (Calculations, Models). **(C)** Initial (pre) and end-of-quarter (post) scores for the Math-Biology Values Instrument. **(D)** Initial (pre) and end-of-quarter (post) scores for CURE survey, Calculations, and Models. Students in both the CURE and comparison groups had very high initial scores for Interest, Utility, Personal Value, and Models, limiting the potential to observe positive changes. Green indicates the initial (pre) survey scores and purple indicates the end-of-quarter (post) scores. Points show the mean and lines show ± the standard error of the mean (SEM) within each group, question, and survey timing. Solid lines show SEM for CURE students, and dotted lines show SEM for comparison students. There are 33 students in the CURE group and 16 students in the comparison group.

Students taking the CURE did not differ significantly from the comparison students in the three constructs assessed by the Math-Biology Values Instrument (Andrews et al., 2017; *p*-values of 0.37, 0.41, and 0.78 for interest, utility, and cost, respectively). We note that both groups increased in both the perceived utility and cost of taking courses that include quantitative biology material (Figures 2A,C). However, it was difficult to assess changes in the Interest and Utility scores, especially for the CURE students, because even the start-of-quarter (pre-course) scores were near saturation (Supplementary Figure S1).

### Gains in Student Attitudes Toward Learning Science

Students in the CURE had significantly more positive changes for the Learning Science construct (*p* = 0.0009). This appears to be largely driven by a more negative end-of-quarter response within the comparison group (Figures 2B,D). Although many CUREs have yielded positive attitudinal outcomes for students (e.g., Brownell et al., 2012, 2015; Jordan et al., 2014; Olimpo et al., 2016; Rodenbusch et al., 2016; Kirkpatrick et al., 2019; Murren et al., 2019), a pattern of more negative responses at the end of a course has been found in previous assessments of student perceptions of science (Adams et al., 2006; Semsar et al., 2011; Perera et al., 2017). Negative changes in attitudes may reflect the impact of other courses, as well as general changes in student morale toward the end of an academic term. Therefore, in the absence of a targeted educational intervention, in some cases the default expectation on a survey may be a slight decline in attitudes from the start to the end of a quarter. The CURE and comparison students did not differ significantly in their changes in Personal Value (*p* = 0.63), although this attribute was difficult to assess because both groups’ pre-course responses were very positive (Figures 2C,D). For the two new questions about the utility of quantitative calculations and mathematical models, CURE students showed more positive changes only for the Calculations question, although neither comparison fell below a *p*-value of 0.05 (Figures 2B,D; *p* = 0.051 and *p* = 0.36 for Calculations and Models, respectively). Similar to the pattern for the Learning Science construct, students in the comparison group gave more negative responses about Calculations at the end of the quarter, while students in the CURE had a small average increase. However, we note that these mean changes in Calculations were small relative to the standard error for each mean. For the Models question, every respondent in both groups selected “Agree” or “Strongly agree” on both the initial and end-of-quarter survey. With such positive initial attitudes, there was limited opportunity for student responses to positively change and neither group showed much average change on this question (Figure 2D).

### Some Students Increased Interest in Future Quantitative Experiences

Because students enrolled in the CURE had very positive initial attitudes toward quantitative biology, we added short-response questions in the 2019 survey for CURE students. We hoped that these would provide complementary insight into how student thinking and attitudes had changed as a result of completing the course. Table 3 summarizes student responses to the initial (pre) question “*Do you have any plans to pursue future courses and/or a career in quantitative biology? Please briefly explain why or why not.”* and the end-of-quarter (post) question “*Has this course changed your plans for pursuing future courses and/or a career in quantitative biology? If so, how?*” These questions were only used in fall 2019 for students enrolled in the CURE course. Six of the 17 respondents expressed an increased interest in pursuing additional coursework or a career in quantitative biology. Two others shared that the course offered some useful clarification about what quantitative biology work entails. Five respondents had a sustained interest; these students expressed an interest in quantitative biology on the initial survey and did not indicate any change in their interest. Finally, four responses were not directly related to the prompts or could not readily be placed into the categories mentioned above.

## Discussion

We have outlined an introductory course-based undergraduate research experience that focuses on building students’ practical laboratory technique and developing quantitative skills for mathematical modeling. Initial assessment of student attitudes during the first 2 years of this course suggest that, relative to a comparison group, students develop more positive attitudes toward the process of learning science, and potentially also see more value to using quantitative calculations in biology (Figure 2). More than one-third of respondents in 2019 also expressed greater interest in taking additional quantitative courses or pursuing future work in quantitative biology (Table 3).

Changes in students’ future quantitative biology course and career plans seemed to be driven in part by an increased interest in or enjoyment of quantitative biology. Among the six students who mentioned a change in their plans, two explicitly discussed increased interest as a factor shaping their future plans (e.g., “The course has definitely made me more interested in quantitative biology.”) and two others mentioned positive feelings about doing quantitative biology (e.g., “I definitely found the passion that I was expecting.”). Some responses also revealed how students might be weighing the relative utility and cost of learning quantitative biology (e.g., “Though math is a challenging and often daunting subject for me, I do believe that it is essential to understanding and applying a bit of it into the science world…”). In future course implementations, post-course student interviews might help reveal how different dimensions of these attitudes interact to shape students’ future academic decision-making.

The Likert-scale assessment of values surrounding the role of math in biology found limited evidence of gains. However, the students enrolled in this course entered with high interest and already believed that quantitative skills were useful in biology, as reflected in the high pre-course scores in the categories of Interest, Utility, Personal Value, and Models (Supplementary Figure S1). This high baseline limited the potential for us to identify gains in these affectual categories.

At the end of the quarter, students in both the CURE and the comparison group perceived a higher cost (e.g., higher workload or lower grades) to taking biology courses that incorporate math. This might be expected, as even students who have a positive, confidence-building experience may develop more realistic expectations about the potential challenges ahead. However, a student’s belief in their ability to succeed at an academic task can affect their academic achievement (Doménech-Betoret et al., 2017), so high perceptions of cost may negatively impact a student’s course outcomes. As we assess larger sample sizes of students who complete both this fall course in microbial culturing as well as the quantitative spring course in comparative genomics, we plan to analyze whether the longer experience over two quarters might produce shifts toward lower perceived costs. Previous work assessing the Genomics Education Partnership CURE (Shaffer et al., 2010) suggests that students perceive greater learning benefits from longer experiences working on their research projects (Shaffer et al., 2014).

In future versions of this course, we hope to include additional activities focused on helping students build their identity as scientists. By design, the course’s focus on student research implicitly places students in the role of research scientists. However, student scientific identity could potentially be developed more explicitly by diversifying the structure of meetings and assignments to promote the kind of informal critical thinking, curiosity, and collaboration that occurs in research labs. Examples might include journal clubs, science coffee chats, poster making and presenting, and academic writing practice. To expand collaboration, one might convert part of each lab into a “lab meeting” in which students discuss with peers and take turns summarizing primary literature or sharing updates on their research (e.g., in the CURE presented by Oufiero, 2019). In addition, we would like to help students understand that failure, mistakes, and repeated iteration of data gathering and analysis are normal parts of scientific inquiry. Although the instructors in this CURE discussed these themes in passing during lecture and lab (Seidel et al., 2015), these goals were not explicit in our lesson planning or assessments.

One logistical challenge for this course was the organization of isolate metadata. Student project ownership may be a foundational source of student benefits from CUREs (Corwin et al., 2018), so we asked students to own their data from sample to final isolate to quantitative growth data. After students struggled to maintain a complete chain of metadata for their samples in 2018, we implemented a Google Sheets system in which all new data were added as new columns to a continually growing classroom document. This became cumbersome by the end of the quarter, but also provided a shared workspace in which students could note each other’s discoveries and feel like a part of a team effort.

Although we focused on salt-dependent growth rates for halophiles, this course could be adapted to other CUREs based around alternative research questions. Other phenotypic investigations might include color production, sugar utilization, halophilic gas vesicle production, or the production of easy-to-spot products like polyhydroxyalkanoates—all of which require a separate set of research techniques scalable to a course timeframe (e.g., colorimetric assays, visible light microscopy, etc.). One could expand on this to culture strains in various growth conditions such as shaking, oxygenation, salinity, pH, nutrient availability, and/or temperature. The most promising alternatives are likely to be those which use a highly selective growth medium, preventing the cultures from being swamped by local contaminants. Our high-salinity media helped reduce contamination problems; extreme temperature, pH, or unusual food sources might be similarly effective.

The skills developed and data created from microbial culturing provide a productive way to engage undergraduate students in course-based research. By combining laboratory skills with growth rate modeling, students learn quantitative skills in a low-stakes environment in which they have ownership over the data they are generating and analyzing. The current version of this quantitative biology CURE emphasizes the growth rates of halophilic microbes, but we expect that this model for course design and implementation can be readily applied to a broad range of organisms and phenotypes.

## Data Availability Statement

The raw data supporting the conclusions of this article will be made available by the authors, without undue reservation, to any qualified researcher.

## Author Contributions

AY, KD, HK, JE, JA, MG, MF, RF, and SA contributed to conception and design of the course. MG, MF, and RF contributed to conception and design of the educational assessment. JA, MF, and RF each served as co-instructors for at least one iteration of the course. HK and SA each served as teaching assistants for one iteration of the course. AY provided technical laboratory support. RF performed the qualitative and statistical analysis. KD, HK, MF, RF, and SA wrote the initial draft of the manuscript. HK, MF, MG, and RF reviewed and edited the initial draft.

## Conflict of Interest

The authors declare that the research was conducted in the absence of any commercial or financial relationships that could be construed as a potential conflict of interest.
