# Supplementary material for: Combining Microbial Culturing With Mathematical Modeling in an Introductory Course-Based Undergraduate Research Experience
Source: Front Microbiol. 2020 Nov 6;11:581903. doi: 10.3389/fmicb.2020.581903 (PMC7674939; doi:10.3389/fmicb.2020.581903)
Supplement: Supplementary file 1 [file Data_Sheet_1.docx]

Combined Supplementary Files (part 1; curricular resources) for “Combining Microbial Culturing with Mathematical Modeling in an Introductory Course-Based Undergraduate Research Experience” by Furrow *et al.*

Table of Contents

[Supplementary File S1: Supplementary Figure S1 2](#_Toc54642162)

[Supplementary File S2: Weekly Course Schedule 3](#_Toc54642163)

[Supplementary File S3: Lab 5 Protocol 5](#_Toc54642164)

[Supplementary File S4: Final Project Guidelines 11](#_Toc54642165)

[Supplementary File S5: Excerpt from Homework 7 -- Modeling a final write-up 13](#_Toc54642166)

# Supplementary File S1: Supplementary Figure S1

**
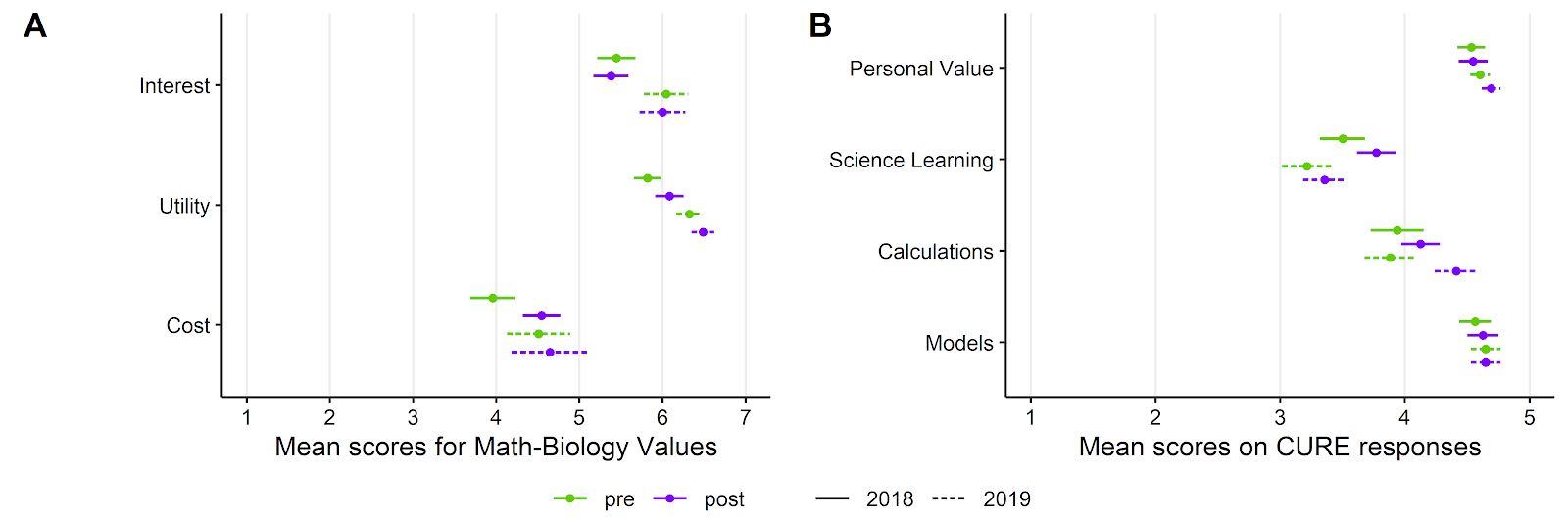
**

**Supplementary Figure S1.** Mean pre- and post-course scores on the Math-Biology Values Instrument (7-point Likert scale) and the CURE survey (5-point Likert scale) for students enrolled in the quantitative biology CURE, separated by year. A) Initial (pre) and end-of-quarter (post) mean scores for the Interest, Utility, and Cost constructs of the Math-Biology Values Instrument. B) Initial (pre) and end-of-quarter (post) mean scores for the Personal Value and Science Learning constructs of the CURE survey, as well for the two additional questions: Calculations and Models. Students in both years had similar changes from pre to post. Green indicates the initial survey scores and purple indicates the end-of-quarter scores. Points show the mean and lines show +/- the standard error of the mean (SEM) within each group, question, and survey timing. Solid lines show SEM for CURE students in 2018, and dotted lines show SEM for CURE students in 2019. There are 16 students in the 2018 CURE group and 17 students in the 2019 CURE group.

# Supplementary File S2: Weekly Course Schedule

BIS 23A: Genome Hunters

Weekly Schedule

| **Week** | **Laboratory skills** | **Quantitative skills** | **Assessment** |
| --- | --- | --- | --- |
| 1 | Lab safety | Troubleshooting and introduction to programming in R | Homework |
| 2 | Pipetting and sterile technique | Quantifying precision and accuracy, calculating using R | Homework |
| 3 | Inoculating cultures | Calculating dilutions for plating liquid cultures | Homework |
| 4 | Plucking colonies and creating pure cultures | Understanding sources of noise and bias in culture-based microbial sampling methods | Homework |
| 5 | Streak plating, initial growth assays | Introduction to models of population growth, step-by-step approach to interpreting a differential equation | Homework |
| 6 | Salt-dependent growth assays and student experiments (e.g. pairwise competitive plating in 2019) | Generating model predictions and comparing with observed data, evaluating quality of fit with adjusted R^2^ and residual plots | Homework |
| 7 | Computational lab – no wet lab this week | Modifying models, explaining the biological meaning of new terms, comparing model fits | Proposing final model |
| 8 | Genomic DNA extraction | Student analysis of growth curves generated from course strains | Homework |
| 9 | Group project work: students proposing and justifying their choice of model to explain growth patterns in course strains. Different groups selecting qualitatively different sets of growth curves from a full set of strains. | | Presentation draft and peer review |
| 10 | Student presentations, including a step-by-step explanation of the chosen model, the method of fitting, the quality of fit, the biological interpretation, and any lingering questions | | Final written report and presentation |

# Supplementary File S3: Lab 5 Protocol

**Lab 5: Preliminary phenotypic characterization of microbial growth**

**Learning Goals. At the end of this lab you will be able to:**

1. Present several potential models and hypotheses to explain patterns of bike distributions on campus,
2. Perform a streak plate to assess the purity of a liquid culture,
3. Explain the underlying measurements of a kinetic growth assay and make predictions about what you expect to see.

##### YOU DO NOT NEED TO WATCH YOUR GROUP MATE DO EVERYTHING OR HELP HOLD THEIR TUBES. FOR THE PRACTICE PART OF THIS ASSIGNMENT DO YOUR OWN PIPETTING. FOR THE PART WHERE YOU BEGIN TO MAKE DILUTIONS FOR YOUR PLATE SPLIT THE WORK BETWEEN YOU AND YOUR PARTNER AND EACH DO YOUR OWN PART. #####

**SAFETY and TRASH MANAGEMENT**

**Chemicals and/or biologicals:** Salt solutions, environmental samples, agar plates, liquid growth media, 70% ethanol

**Materials:** *Disposable pipet tips; 14 ml Falcon tubes, 1.5/2mL tubes, agar plates; 96-well plates*

**Equipment:** *Alcohol lamps, pipets, inoculation spreader, incubator; Tecan Plate Reader*

**Required PPE:** Lab coat, gloves and eye glasses while the wet-work is happening.

**Trash and disposal:**

- *Pipet tips and serological tips: Dispose of these in marked plastic lined collection bins on each lab bench*
- *1.5/2.0 mL tubes: discard tubes and any solution therein in the plastic lined collection bins on each lab bench*
- *Left over microbial starter solutions: leave these capped on the bench, the TA will clean these after class*

**Hazards:** *Ethanol is flammable - use small volumes and keep away from ignition source;* ***FLAMES - LONG HAIR SHOULD BE TIED BACK BEFORE STARTING****;* *ensure that all tubes are fully closed before mixing or using Vortexer or placing them in the incubator; Ensure that centrifuges are balanced; at 100g/L NaCl and above, the chances of isolating anything pathogenic are extraordinarily slim - nevertheless, learn to treat microbial cultures with respect and clean up spills with bleach and paper towels, put soiled gloves in the plastic lined collection bins, wash your hands before/after lab.*

**Possible Nuisances:** *Sometimes, microbial cultures stink. That’s ok! If it’s really bothering you, say something to the instructor.*

**Overview**

**Previously**:

Last week you started pure cultures by plucking individual colonies from your plates. You also compared colony morphology across the class, noting the diversity of organisms we have collectively isolated.

**This week**:

We want to start quantitatively characterizing a phenotype of the organisms you’ve isolated. We’re going to start by measuring growth rates of these organisms in the isolation media. Later we will select some organisms for further study and repeat the phenotypic characterization of growth on a more limited set of species but across more diverse growth conditions.

**You will again be working in groups of 2 but will each have separate responsibilities. Group up!**

Each group should first make sure that you have or have easy access to the following items:

| ***Tools and disposables*** | ***Equipment*** | ***Reagents*** |
| --- | --- | --- |
| 1 - 1000 µL Pipetter  1 - 100 µL Pipetter or 200 µL Pipetter  1 - 10 µL Pipetter  1 - box of 1000 µL Tips  1 - box of 100 µL Tips  1 - box of 10 µL Tips  1 - box of 1.5 mL tubes  4 - 10 µL inoculating loops | Access to:  37C shaking incubator  37C incubator (not shaking)  Refrigerator  Proper adapters for shaking incubator  TECAN Microplate reader | Agar plates (150g/L & 250g/L)  2 - liquid halophile media (150g/L & 250g/L) |

**Classroom Activity with Bike Data**

We will build on Tuesday’s lecture to do some model thinking with the bike data that you gathered last week. Instructors will guide the activity.

**Continued Observations of Agar Plates**

In the following, we continue to characterize the growth (or lack thereof) on agar plates. If you have any new plates with growth, record your observations below.

Plate notes: (Include the sample details, then describe the colonies on the plate.)

**Observations of Pure Liquid Cultures**

Most of you started some liquid cultures of single isolates. For each of your cultures (you may only have one) write down the basic details. ***Today we will also assign a unique ID number to each isolate, to streamline labeling for the rest of the quarter.***

**SAMPLE 1**

Name/source/isolation media: _____________________________________________

Pure culture media & ID number: ___________________________________________

Please write your observations about this liquid culture in the space below.

**SAMPLE 2 (if relevant)**

Name/source/isolation media: _____________________________________________

Pure culture media & ID number: ___________________________________________

Please write your observations about this liquid culture in the space below.

**Pure Culture Assessment**

The liquid cultures we created are theoretically pure cultures of isolates from different salt and environmental samples. We will assess purity by making a streak plate from this culture. Though we will not be able to confirm with this method alone, it is a good sign to only see a uniform profile of colonies in this streak plate. As seen in the figure below, streak plates are prepared by sequentially streaking the loop (or pipette tip) gently back and forth at 4 distinct regions of the plate, which in effect, helps one obtain isolated colonies.


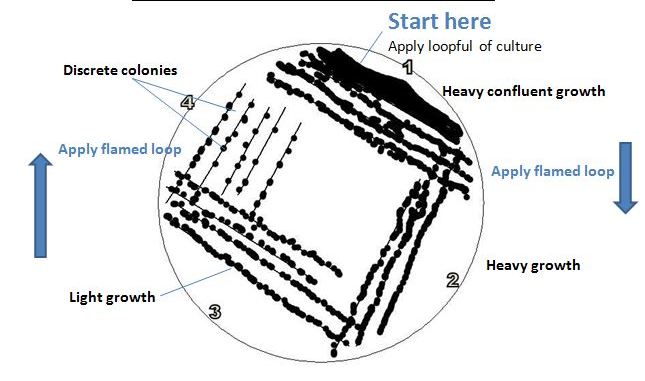


**Create your own streak plates using your pure cultures.**

**Collecting Preliminary Growth Data**

Each of you should have started a liquid culture of at least one isolated sample (some of you will have more than one sample). To save time, your instructor will have already pre-determined the OD_700_ of each sample on the day of the lab.

Given each your samples’ OD_700_, you will need to calculate appropriate dilutions of your samples to start the growth cultures.

**The target OD_700_ for the starter cultures is 0.05. THIS IS WHAT YOU ARE TRYING TO MAKE WITH EACH OF YOUR SAMPLES. DILUTIONS ARE TO BE DONE IN THE ISOLATION MEDIUM**

You will want to make a total of ~500µl of sample at a final OD of 0.05. Write a plan how you intend to meet this goal for each sample in the space below and have your instructor review the plan before starting.

**Hint: try to finish with by having a 500 µL volume (or slightly less) in a 1.5 mL tube.

**Loading a 96-well plate**

You will need to load your samples in a 96-well plate for growth experiments. **DESIGNATE ONE PERSON FROM EACH GROUP OF 4 TO LOAD THE PLATE - THEY WILL LOAD THE PLATE.**

The designated plate loader will load 200µl of diluted sample into appropriate wells. Each student is assigned one row on a plate in columns 2 through 7. Each isolate sample will be loaded twice per plate according to the figure below. Columns 10-11 are reserved for the instructors.

Write down your plate number and circle the row that you have designated for your samples.

Plate Number:______________


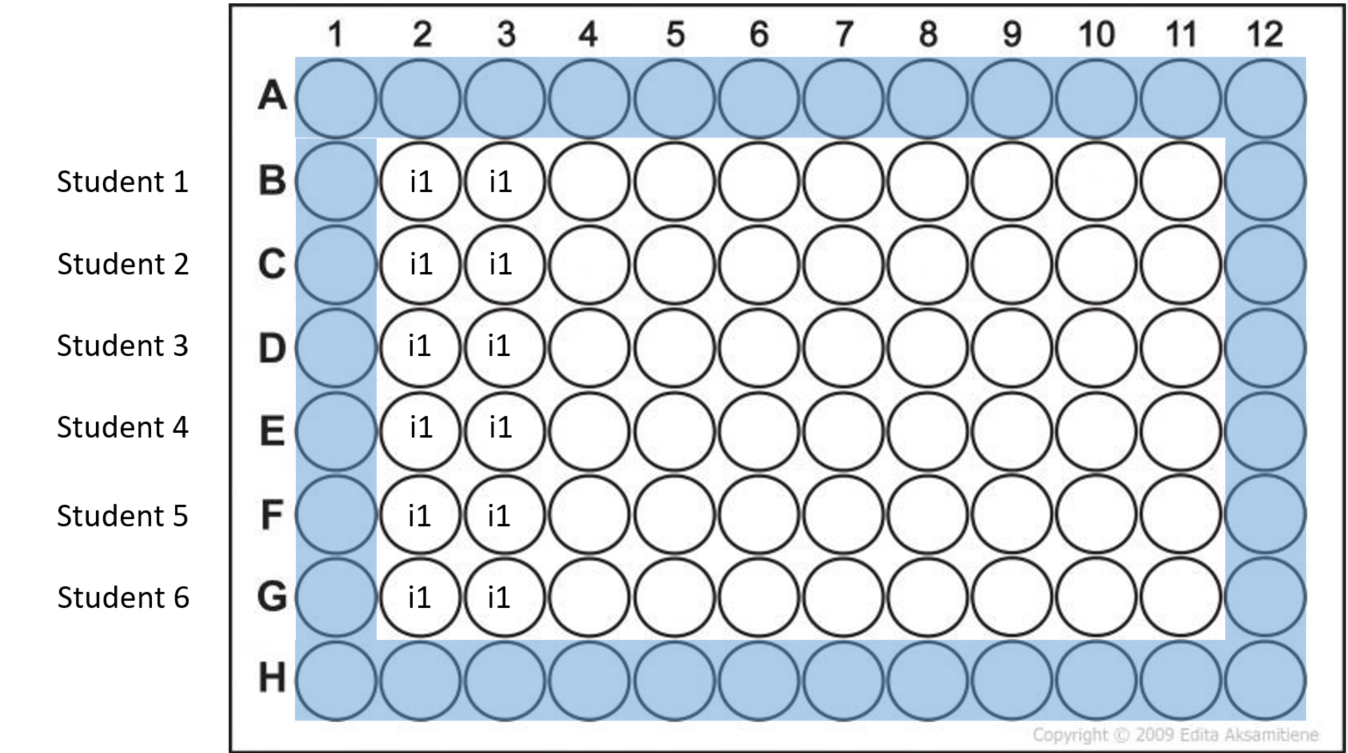


**Typical 96-well plate format:** A typical 96-well plate has 96 independent wells organized into 8-rows and 12-columns. We can use these plates to conduct many quantitative growth experiments at the same time. The shaded area will be “sacrificed” and filled with media to provide a buffer against drying. This week, each student gets part of a row. Your isolate will be loaded in duplicate.

**Isolate 1**

Name/source/isolation media: _____________________________________________

Pure culture media: _____________________________________________________

Exact locations on plate (Row letter and column numbers):_______________________

**Isolate 2 (only if discussed with instructor)**

Name/source/isolation media: _____________________________________________

Pure culture media: _____________________________________________________

Exact locations on plate (Row letter and column numbers):_______________________

**Reflections/Predictions**

In the space below write some reflections on (these will go into your next lab write-up):

1. You’ve started some new liquid cultures that will grow for five days. This growth will be monitored at regular intervals using the Tecan plate reader by measuring light scattering at 700nm. Write down and/or sketch what you predict to see at the end of this period from the samples that you’ve loaded in the plate reader.
2. Think about what other phenotypes you might want to measure quantitatively. Suggest how you might make those measurements. This doesn’t need to be detailed - a general scheme will do.
3. Propose a design for an experiment to measure growth rate that would involve plating portions of liquid cultures on agar plates rather than measuring turbidity of the liquid cultures themselves. Use the internet as a resource if necessary.

# Supplementary File S4: Final Project Guidelines

**BIS23A FQ2019 – Final Project Guidelines**

For your final project, you will work in groups of 2-3 people to conduct deeper research, both biological and with quantitative modeling, write an individual report, and present on the topics. Presentations will take place during lab on **Thursday, December 5^th^**, and the report will be due on **Friday, December 13^th^ at 10am.** The final project is worth a total of 25% of your grade, but you will get lots of feedback along the way as you work.

**Individual final report (15 points)**

The report should be 6-7 pages, and include a code appendix (not part of page count) with all R code used for the modeling component. This sounds long, but we will use lab time and homework assignments to guide you through gradual progress in the latter weeks of the course. The final report is an individual project, although you will have a shared topic and should be analyzing the same model as the rest of your group. The final report should include the following components:

- **6 points – A discussion of halophile-specific academic research (2-3 pages), including:**
  - **Introduction** – why are people doing this research, how does it connect to what people already know and they hope to learn?
  - **Relevance to BIS 23** – how does this relate to the topics covered in this course?
  - **Study details (longest section)** – for at least two peer-reviewed academic studies, what was their methodology? What did they study, what tools did they use, what did their results look like, and what conclusions did they draw from them?
  - **Future direction** – think of 2 research ideas that involve your topic; why is your research idea important and how does your topic support your research ideas?
  - **Conclusion** – please provide closing remarks about your topic; feel free to provide personal thoughts and opinions regarding this topic (what personally intrigues you, what concepts were more difficult to understand, how did you address difficulties learning about your topics, etc.)
- **6 points – Analysis and refinement of a growth rate model (2-3 pages), including:**
  - **Introduction** – what is the basic logic for the logistic growth model, and why is it useful across a range of topics?
  - **Logic of your model –** what kinds of modification(s) did you make to get to your final model? What is the biological basis of the changes you made?
  - **Details of your model (longer section)** – what do the equations look like? What is the initial and long term behavior of the model (e.g. for logistic growth, it looks linear for small populations and then plateaus as you reach a carrying capacity)
  - **Your technical analysis (longer section)** – how did you fit this model to real data? Did it improve the fit, compared to a logistic growth model? How do you know? Calculate relevant statistics to demonstrate the quality of this fit.
  - **Your conclusions** – discuss what this model tells you about the biology of our study system (halophiles in a closed, liquid system). How might this model be useful for researchers? What additional data would help you distinguish this model from other reasonable hypotheses about microbial growth patterns? What would you hope to study next?
  - **Code appendix** – should include clearly commented code with all analyses and plots performed in R. *This does not count towards the word limit.*
- **3 points – Your personal reflections on the course (1 page), including:**
  - **Ideas for modifications –** What would you like to see done in the future? We performed growth assays but what other phenotypes might we want to measure quantitatively? Suggest how we might make those measurements. Include hand-drawn or computer-generated figures if beneficial in explaining.
  - **Reflection on learning –** What was challenging about the course? What was most fun? What do you feel you learned well, and what is still confusing? Please share other thoughts on your learning process here as well. This prompt is left open-ended on purpose.

**Presentation (10 points)**

Each presentation is expected to be approximately 25-30 minutes in duration, including time at the end of the presentation for questions. The topics have already been assigned, and will be finalized in Lab 6.

The structure of the presentations are similar to the final project, although you will need to fully reach consensus as a group on exactly what you cover, and who is presenting for which sections.

- **Introduction to research topic** – why are people doing this research, how does it connect to what people already know and they hope to learn?
- **Relevance to BIS 23** – how does this relate to the topics covered in this course?
- **Study details (longer section)** – focus on a single academic research study, and explain it in detail. What did they study, and how? What did they find, and why is it interesting/relevant?
- **Future direction** – think of 2 research ideas that involve your topic; why is your research idea important and how does your topic support your research ideas?
- **Introduction to modeling** – very brief discussion of the growth rate data we gathered in this course
- **Logic and details of your model –** what kinds of modification(s) did you make to get to your final model? What is the biological basis of the changes you made? What do the equations look like?
- **Your technical analysis (longer section)** – how did you fit this model to real data? Did it improve the fit, compared to a logistic growth model? How do you know? Calculate relevant statistics to demonstrate the quality of this fit.
- **Your modeling conclusions** – discuss what this model tells you about the biology of our study system (halophiles in a closed, liquid system)? How might this model be useful for researchers? What would you hope to study next?
- **Final conclusions –** please provide closing remarks about your project; feel free to provide personal thoughts and opinions regarding this topic (what personally intrigues you, what concepts were more difficult to understand, how did you address difficulties learning about your topics, etc.)

We expect participation and communication from all members of the team – this may take the form of out-of-class meetings, group emails/chats, collaborative platforms (Google Docs/Presentations), etc.

We will share detailed rubrics in future weeks as you begin work on the two project components.

# Supplementary File S5: Excerpt from Homework 7 -- Modeling a final write-up

**[Notes for instructors. Below we share part of a course homework. This includes a sample write-up of a final model report and prompts for students to write some of their own work in a similar style. The sample had strengths and weaknesses that students discussed during the class session after this homework was due. To highlight some topics that we discussed in that session, we also include annotations in red bold text.]**

**Mosquito growth tutorial and your application to your own model**

Your instructors want to demonstrate an approach to creating and analyzing a model, then writing it up. But we don’t want to use data that are too similar to a microbial growth curve. Instead, we focus here on a study of mosquito growth in some artificial ponds in a town near Oxford, England. If you are curious, the full data and paper are here: <https://parasitesandvectors.biomedcentral.com/articles/10.1186/s13071-019-3321-2>

The data in this case are the counts of mosquitos caught in traps over the course of one season. We will focus only on counts of adults of a single species, *Culex pipiens*, which is the most common species in most of North America and across the northern hemisphere.

Now we will go through the different components of the modeling portion of the final report, demonstrating how we might approach them with our data. Each of these sections are a briefer version of the real thing, but will offer an example for how to organize things.

**Introduction**

Many insect species reproduce rapidly, only experiencing resource limitation after rising to high population sizes. The logistic growth model explicitly incorporates both exponential growth and a carrying capacity, predicting a sigmoid curve that appears initially exponential before leveling off to a fixed population size. However, in practice, many other factors can influence growth and mortality rates. For example, for mosquitos, seasonal effects such as temperature can influence both survival and reproduction. In many regions, extended cold weather kills most mosquitoes, while a small fraction of adults (and a larger fraction of eggs) survive in dormancy until warm weather returns. The count data we analyze (Figure 1) reflects this population decline at the onset of cooler fall temperatures. To model this, we will modify the growth rate of an exponential growth model to incorporate season-dependent effects.

**[Strengths of this section: 1) it provided brief context for logistic growth modeling, 2) it related an environmental/biological phenomenon (seasonal temperature) to a prediction about population sizes, 3) it included a clear plot of the full, raw data (although this could just be a reference to a figure that also includes the model fit… it doesn’t need to be duplicated here). Weaknesses: 1) for reproducibility, the data collection process should be explained in more detail, 2) a brief explanation of the study species would ensure that readers are on the same page (although most readers will know what a mosquito is).]**


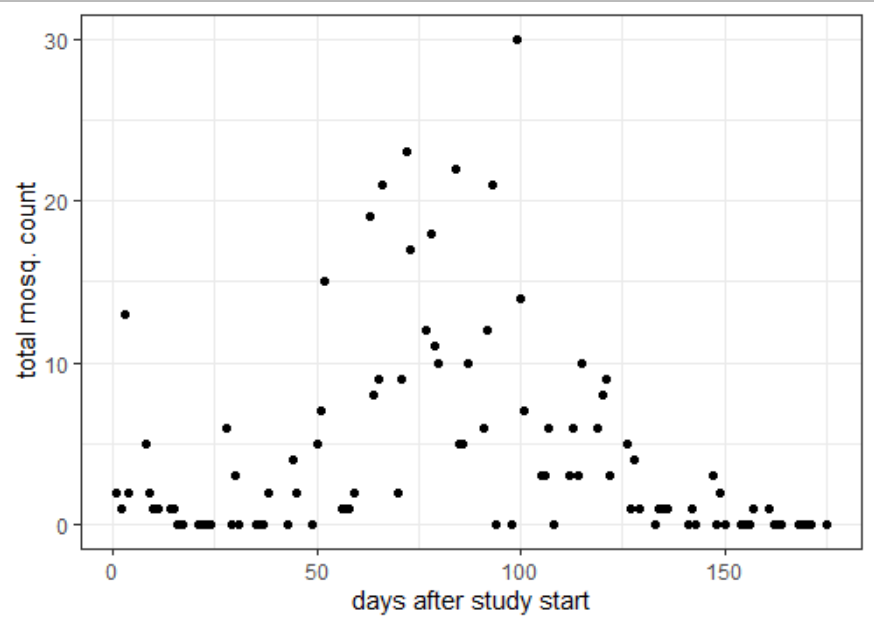


Figure 1. Daily counts of adult C. pipiens, starting from day 1 of the study (14 Apr. 2015)

**The model**

This model is based on a simple model of exponential growth through time, with a modification of the growth rate *r* to include a periodic seasonal effect. The equation for our growth model is:

$$N\left( t \right)=N_{0}e^{rt*\sin\left( \frac{\left[ t-\tau\right]2\pi}{365} \right)}$$

The model is nearly identical to exponential growth with rate *r*, but the growth is now impacted by an additional sinusoidal term. This term allows the growth to be negative (for example, during cold conditions), leading to population declines during a winter period. Because the onset of good or poor growth conditions does not need to line up with the start of the observations, the term $\tau$ represents a time lag term. When $t\geq\tau$, the sine term becomes positive (until 182.5 days later when the term becomes negative again). The factor $\frac{2\pi}{365}$ is used to scale from radians (where the sine wave repeats over $2\pi$ radians) to days (the units used in our study). This allows the periodic function to be identical each year, to align with annual seasonal patterns.

At time $t=0$, the model predicts an initial population value of $N_{0}$. However, the long-term predictions of the model beyond a single year may be biologically unrealistic. Although the periodic sine term will be identical each year, as *t* continues to increase, the *rt* term will become very large, leading to predictions of extremely high growth in future years (see Figure 2). For that reason, this model is likely to be useful only for modeling a single warm season’s growth.

**[This section is meant to show some of the crucial components that should be explained when presenting a model, including the equation, a comparison with related models, and a description of the biological meaning and mathematical impact of each term. It’s great that the writer recognizes the limitations of this model. However, this exploding periodic term is a fundamental flaw. The periodicity might have been better including as an additive term, so that the effect is not magnified as time progresses. During the discussion, we asked students to brainstorm alternative ways to introduce periodicity into a model like this.]**


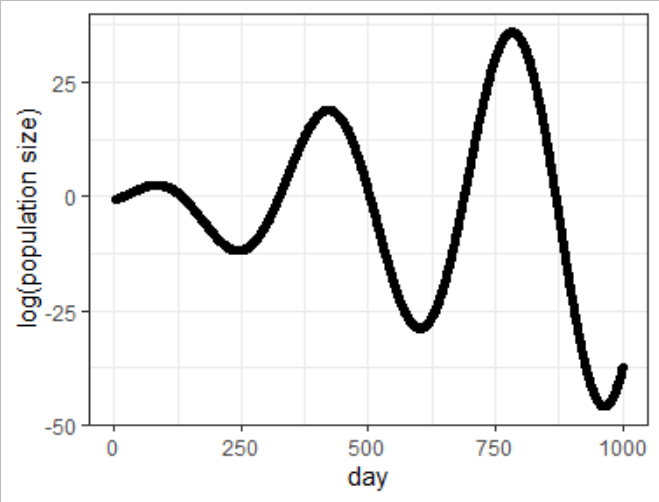


Figure 2. Population size predicted over several years with this model (tau = -43, r = 0.47, N0 = 0.49). Population size is plotted on a log scale to show the fluctuations within a reasonable range.

**Model results and analysis**

To fit this model to the raw mosquito count data, we apply non-linear least squares regression. This technique uses numerical methods to find model parameter values that minimize the residual sum of squares. To perform this regression, we use the function nlsLM() from the package “minpack.lm”. We compared our model fit with the best fit of the logistic growth model. Because both models have 3 parameters, we report and compare the R^2^ values to assess quality of fit. *[Note to students, you’ll want to use adjusted R^2^ to compare models if your model and the baseline comparison, e.g. logistic growth, have a different number of parameters.]*

**[Although we don’t ask for a full explanation of the non-linear least squares method in the paper, we expect students to be able to outline the logic of this step-wise process when asked in a one-on-one situation.]**

Figure 3 illustrates the raw data, overlaid with the model best fit for our model and the logistic growth model. Our periodic model captures both the initial growth, as well as the later decline in mosquito counts, whereas the logistic growth model consistently underpredicts counts at the seasonal peak, then overpredicts counts at the end of the study. Figure 4 demonstrates the residuals for our model fit. We can see that the variability in the residuals is not consistent, with higher variance during peak season when counts varied more. However, there are no periods with residuals consistently biased to be above or below zero. This suggests that the model may be capturing some of the fundamental driving forces behind the pattern of rise and fall in mosquito counts. The model R^2^ values for the periodic and logistic models are 0.42 and 0.06, respectively. Although this demonstrates that our model fits these data substantially better than a logistic growth model, a considerable fraction of the variation is still unexplained. Some of this may be due to the inherent variation in small-scale sampling of a large insect population, but some may be due to other biological processes that could be modeled.

**[This write-up makes good use of figures to compare with the best-fitting logistic model, and the inclusion of a residual plot is crucial to show whether the model is systemically biased at certain times. We emphasized that one of the first things you should do after model fitting is plot the model fit overlaid on the raw data, as well as plot the residuals. It might have been more persuasive to have Figure 4 show the residuals for both models – that would have particularly highlighted the issues with a logistic model for these data. The write-up also does a good job of precisely discussing R^2^ as a proportion of variation in the data, using statistical language to reflect on the variation that the model explains and the variation that remains unexplained.]**


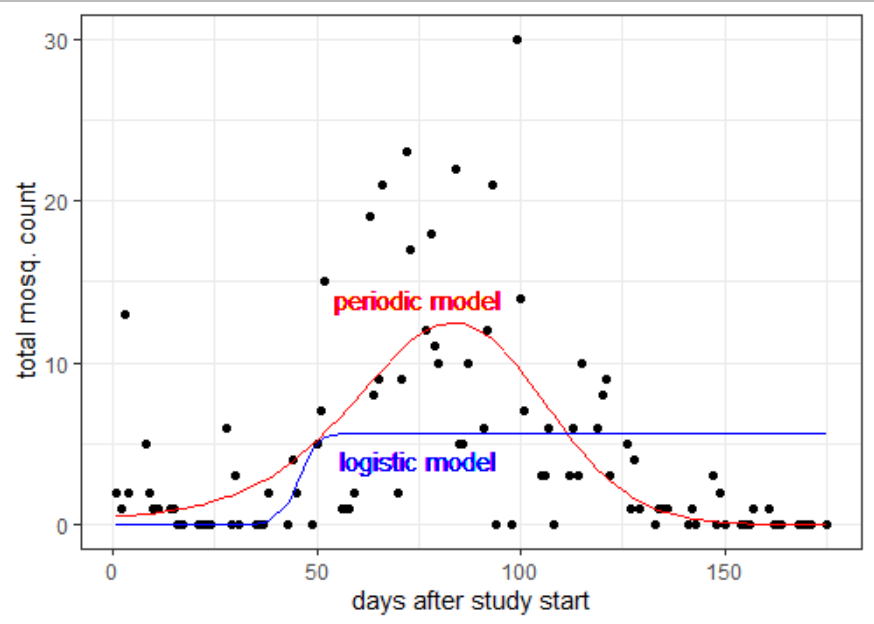


Figure 3. Best fits for logistic and periodic models.


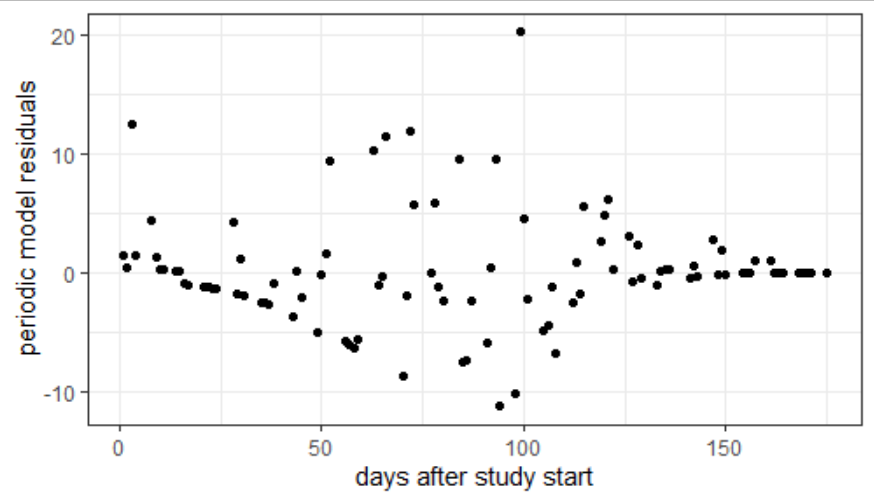


Figure 4. Residuals for our periodic model.

**Conclusion**

Overall, our model fits these data fairly well, and suggests that an underlying periodic effect on growth rate may explain the rise and fall in mosquito counts over a summer season. We consider this periodic effect to represent a potential impact of temperature on mosquito survival and reproduction. With that in mind, the value of parameter$\tau$ offers insight into precisely when conditions might become favorable to insect growth. Using these data, the best fit for $\tau$ was -43, suggesting that initial population growth become positive about 43 days before mosquito rose to detectable numbers. This modeling could inform best practices for the timing of insecticide spraying or other insect control numbers. With a changing climate, we might expect changes in seasonal temperatures to shift the onset of growth even earlier in time, and this model could be used to predict peak mosquito numbers in different temperature scenarios.

**[This paragraph uses careful language to identify the insights that the model can offer without assuming that the model is “correct”. In this course we frequently emphasized that models are, at best, useful approximations that allow you to gain new insight about a system, and we wanted students to use similarly cautious language for their write-ups. The connection to the growth timing and tau parameter was an interesting way to connect to broader global issues with seasonal temperatures. However, the initial mention of the -43 value might have been better placed in the results section.]**

However, we note that the model is limited in scope. It does not make predictions effectively beyond a single growth season (e.g. Figure 2), and artificially imposes that growth must be negative for exactly half of each year (due to the features of a sine curve). To more effectively model growth over multiple years, we would need insect seasonal growth data over multiple years, and would likely need to adjust the model. To address the length of the periods of positive or negative growth, an additional parameter could adjust the sine curve up or down. This would allow the model to be applied in a broader range of regions that have a differing seasonal temperature regimes. In addition, the precise shape of the periodic function could be changed to be steeper or have more complex periodic structure through the combination of multiple periodic functions of different period length. However, we appreciate the simplicity of this model and the interpretability of the parameters.

**[We highlighted that a reflection on model limitations is a critical part of the discussion in a model analysis. Similarly, the prompt for this final report asked students to identify additional data that would be useful for validating a model and/or comparing it with other potentially useful models. We view this as one of the crucial points of a model: using initial results to inspire further data collection that can help demonstrate where the model captures the dynamics of a system well and where the model does not. This paragraph could be strengthened by adding greater details on future data collection. Growth data over multiple years would be great, but the discussion of additional parameters here also seems to call for data from different latitudes and temperature regimes. That might be particularly valuable for understanding exactly how temperature is impacting growth rate, so it could be made more explicit in the text.]**

**Part A. Write your own model details section**

Refer to the final project prompt and the model write-up above. You will now get started drafting parts of your final report, using your group’s model/data. Focus on the section that was labeled above as **The model**. Your task here is to present a model equation, explain the meaning of the parameters, and discuss some of the general behavior of the model.

***Your response here. (2-3 substantive paragraphs)***

**Part B. Write your own model conclusion section**

Refer to the final project prompt and the model above. Focus on the section that was labeled above as **Conclusion**. If you haven’t finalized your model fits, some of this might still be tentative. But it will get you practice thinking about what works and what doesn’t with this model, what you can learn from it, what additional data you might want, and what you might study/model next.

***Your response here. (2-3 substantive paragraphs)***
